# Thrombus Imaging Using 3D Printed Middle Cerebral Artery Model and Preclinical Imaging Techniques: Application to Thrombus Targeting and Thrombolytic Studies

**DOI:** 10.3390/pharmaceutics12121207

**Published:** 2020-12-12

**Authors:** Andrea Vítečková Wünschová, Adam Novobilský, Jana Hložková, Peter Scheer, Hana Petroková, Radovan Jiřík, Pavel Kulich, Eliška Bartheldyová, František Hubatka, Vladimír Jonas, Robert Mikulík, Petr Malý, Jaroslav Turánek, Josef Mašek

**Affiliations:** 1 Department of Pharmacology and Toxicology, Veterinary Research Institute, Hudcova 296/70, 621 00 Brno, Czech Republic; viteckova@vri.cz (A.V.W.); novobilsky@vri.cz (A.N.); kulich@vri.cz (P.K.); EliskaBar@seznam.cz (E.B.); fhubatka@seznam.cz (F.H.); andreaww@seznam.cz (V.J.); 2 Department of Pharmacology and Toxicology, Masaryk University, Žerotínovo náměstí 617/9, 601 77 Brno, Czech Republic; jana.hlozkova.ro@gmail.com (J.H.); scheerpet@gmail.com (P.S.); 3 Stroke Research Program, International Clinical Research Center St. Anne’s University Hospital, Pekařská 53, 656 91 Brno, Czech Republic; mikulik@hotmail.com; 4 Laboratory of Ligand Engineering, Institute of Biotechnology of the Czech Academy of Sciences, v.v.i., BIOCEV Research Center, Průmyslová 595, 252 50 Vestec, Czech Republic; Hana.Petrokova@ibt.cas.cz (H.P.); malyp@ibt.cas.cz (P.M.); 5 Institute of Scientific Instruments of the Czech Academy of Sciences, Královopolská 147, 612 64 Brno, Czech Republic; jirik@isibrnol.com; 6 Institute of Physics, Czech Academy of Sciences, Na Slovance 1999/2, 182 21 Prague, Czech Republic

**Keywords:** 3D printing, thrombus imaging, fibrin targeting, thrombolysis, MRI, microCT, fluorescence imaging, rtPA, MCAO

## Abstract

Diseases with the highest burden for society such as stroke, myocardial infarction, pulmonary embolism, and others are due to blood clots. Preclinical and clinical techniques to study blood clots are important tools for translational research of new diagnostic and therapeutic modalities that target blood clots. In this study, we employed a three-dimensional (3D) printed middle cerebral artery model to image clots under flow conditions using preclinical imaging techniques including fluorescent whole-body imaging, magnetic resonance imaging (MRI), and computed X-ray microtomography (microCT). Both liposome-based, fibrin-targeted, and non-targeted contrast agents were proven to provide a sufficient signal for clot imaging within the model under flow conditions. The application of the model for clot targeting studies and thrombolytic studies using preclinical imaging techniques is shown here. For the first time, a novel method of thrombus labeling utilizing barium sulphate (Micropaque^®^) is presented here as an example of successfully employed contrast agents for in vitro experiments evaluating the time-course of thrombolysis and thus the efficacy of a thrombolytic drug, recombinant tissue plasminogen activator (rtPA). Finally, the proof-of-concept of in vivo clot imaging in a middle cerebral artery occlusion (MCAO) rat model using barium sulphate-labelled clots is presented, confirming the great potential of such an approach to make experiments comparable between in vitro and in vivo models, finally leading to a reduction in animals needed.

## 1. Introduction

Thromboembolic diseases such as acute ischemic strokes (AIS), acute myocardial infarction and pulmonary embolisms are the leading causes of mortality and morbidity worldwide [1,2]. For AIS, reperfusion therapy is the only approved treatment and includes thrombolysis using recombinant tissue plasminogen activator (rtPA) and/or mechanical thrombectomy [3]. However, due to rtPA infusion’s time limitations and the occurrence of bleeding-associated adverse effects in AIS patients, novel thrombolytic drugs are needed [4]. Therefore, there is a need to have clinically relevant in vitro and in vivo models that allow the study of imaging and lysis of blood clots.

Three-dimensional (3D) printing is an essential novel tool for rapid prototyping in biomedical research and technology development, where it has been used as a substitute standard subtractive manufacturing process. In addition, 3D printing is capable of making complex 3D objects that are impossible to make by standard approaches [5].

3D printing allows in vitro models of geometrically complex organs to be prepared, such as vasculature trees, including pathological vascular defects. Importantly, it allows human biological material to be used (e.g., human blood thrombus, plasma, human cells, etc.). This gives an advantage over the pitfalls of animal models, which have arisen from differences in vasculature system physiology and anatomy between humans and animals [6]. Furthermore, in vitro vasculature models allow quick, cheap, and reproducible studies with a reduced number of animals necessary for the experiments.

Imaging techniques such as computed tomography (CT) and magnetic resonance imaging (MRI) are commonly used to visualize the effects of thrombi in patients [7] and in preclinical animal models; however, such imaging is indirect and visualizes the obstruction of blood flow rather than the thrombus. The strategy of direct thrombus targeting using nanoparticle-based targeted contrast agents to the thrombus is a favorable approach for thrombus precise detection and localization. This approach, in principle, can enable exact thrombus size measurement and efficient thrombolytic therapy assessment in both preclinical and clinical studies [8,9]. The possible application of nanoliposomal carriers as theranostics for thrombus treatment was reviewed recently [10].

Developing novel thrombolytic therapeutics requires valid and robust methods to prove their efficacy. In vitro models of cerebral arteries enable new potential thrombolytic compound screenings, which can be further tested using in vivo animal models. In vitro flow models have been used to study thrombus-related events, mainly thrombolysis [11,12,13,14,15,16] or thrombus trajectories [17], over several decades. These in vitro arterial models were produced by various materials and techniques, from simple straight plastic tubes [18] to anatomically precise three-dimensional (3D) printed models obtained by the lost wax method [8,17].

In this study, we have developed a simplified 3D printed model of a middle cerebral artery (MCA) and validated this model by showing its potential for clot imaging. The model was flexible and transparent, allowing the applicability of all the main imaging techniques used in pre-clinical research including CT, MRI, and whole-body fluorescence imaging (FLI) for contrast-labelled clots imaging. Both clinically relevant approaches—diagnostic and therapy approaches—were studied in this work and shown to be feasible to use the MCA 3D printed model and clot imaging techniques to perform (i) direct imaging of the clot visualized by circulating fibrin-targeted liposomes, and (ii) imaging of contrast-pre-labelled clots enabling precise clot size quantification for in vitro thrombolytic studies. Moreover, barium sulphate-pre-labelled clots were shown to be of sufficient intensity of signal for both in vitro and in vivo computed X-ray microtomography (microCT) imaging, making this strategy applicable for fibrinolytic studies.

## 2. Materials and Methods

**Figure 1:**
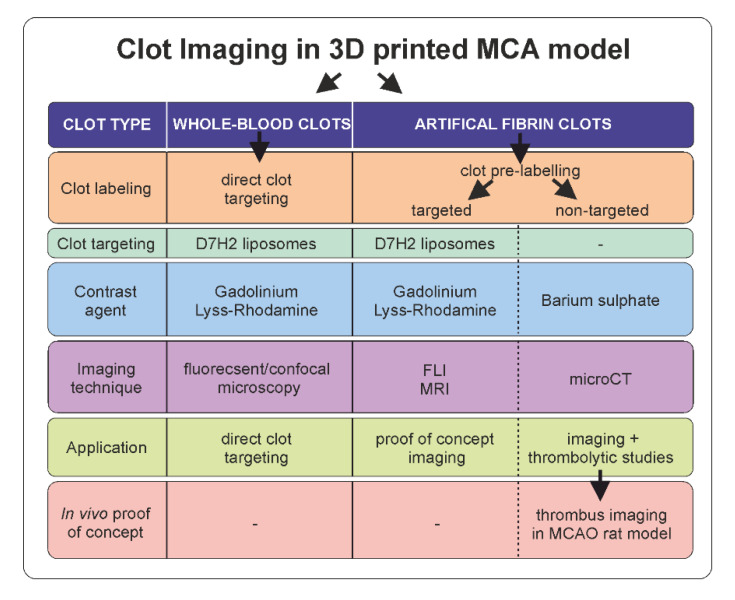
Workflow of clot imaging experimental set up using three-dimensional (3D) printed middle cerebral artery (MCA) model.

This is a study to develop a clinically relevant in vitro model and to validate this model for the purpose of clot imaging. Firstly, a middle cerebral artery (MCA) model was printed using a 3D printer and transformed into a silicone MCA. Model development and validation were conducted in several phases: (i) quality assessment of the silicone MCA model (Scanning electron microscopy (SEM), atomic force microscopy (AFM), and microCT), (ii) ability of the model to lodge the clot (simulation of thromboembolic stroke using the in vitro MCA model), (iii) suitability of the model for clot visualization (in vitro clot visualization in the silicone MCA model using targeted D7H2 liposomes and using preclinical imaging techniques, (iv) suitability of the model for direct clot targeting studies, (v) suitability of the model to monitor lysis of the clot (monitoring of thrombolysis under flow conditions in the MCA model using microCT). In addition, the contrasting potential of barium-labelled fibrin clots for microCT was tested in vivo as a proof-of-concept study. Workflow of clot imaging experimental set up is documented in Figure 1.

### 2.1. 3D Printing of Stenotic Middle Cerebral Artery Model

A simplified virtual middle cerebral artery (MCA) model with bifurcation (2 branches) was designed using Fusion 360 (Autodesk) with an anatomy resembling an average MCA diameter of 3.1 mm, with branches after bifurcation of 2.9 and 1.4 mm. A stenosis was incorporated into the 2.9 mm branch. The “.stl” file was transformed by Prusa 3D Slic 3r MK2 into “gcode“, readable with a Prusa i3MK2 3D printer (Prusa Research, Czech Republic).

In vitro models were prepared by 3D printing the MCA lumen from butenediol vinyl alcohol (BVOH) filament soluble support (Verbatim/Mitsubishi Chemical Holdings Group, Tokyo, Japan). After drying, the model was covered with silicone polydimethylsiloxane, Sylgard^®^ 184 Elastomer Kit (Dow Corning, Midland, MI, USA) (PDMS). The silicone was vacuumed for 20 min and allowed to harden at 25 °C for 48 h. The model was cut into a final shape and the 3D printed lumen was dissolved in distilled water.

### 2.2. Quality Assessment of 3D Printed MCA Model

#### 2.2.1. CT Scans of Models

In vitro silicone MCA models (*n* = 3) were scanned using a microCT Skyscan 1276 (Bruker microCT, Kontich, Belgium) in step and shoot scanning mode and an oversize scan (*n* = 3) at a voltage of 100 kV and current of 200 μA for 541 ms (exposure time) with a central camera position using an aluminum/copper filter, camera binning 2016 × 1344, image pixel size 10.5 μm, rotation step of 0.1 degrees and rotation over 180 degrees. A back-projection dataset of 5422 slices was then reconstructed using InstaRecon software (Bruker microCT, Kontich, Belgium) with a 2.0 post alignment correction, no ring artifact correction, and no beam hardening reduction. The reconstructed dataset was processed using CTan software, where histogram thresholding was set up and the region of interest manually selected. Image thresholding range was 0–50 to visualize the arteria lumen filled with air. Thereafter, the image dataset was saved as a bitmap and 3D volume rendering visualization was performed in CTvox software (Bruker microCT, Kontich, Belgium).

#### 2.2.2. Scanning Electron Microscopy (SEM)

The inner part of the MCA model’s silicone surface morphology was observed using a scanning electron microscope, Hitachi SU8010, Tokyo, Japan. Prior to observation, the surface was sputter coated with Platinum/palladium and the structures of the surface and artifacts were observed at different parts of the model’s inner surface.

#### 2.2.3. Atomic Force Microscopy

Parts of the silicone MCA were attached onto a microscopic slide and placed under an atomic force microscope, Nanowizard 4 (Bruker Nano Surfaces Division, Karlsruhe, Germany). A PPP-FMAuD silicon probe (Nanosensors) with a nominal resonant frequency of 75 kHz and nominal spring constant of 2.8 N/m was used for the measurements. The selected parts of the model’s inner surface were scanned in tapping mode at a scan rate of 0.5 Hz. The size of scans was 20 × 20 µm. All of the images were processed in Gwyddion 2.34 (Czech Metrology Institute, Brno, Czech Republic).

### 2.3. Clot Preparation

#### 2.3.1. Whole Blood Clot

Whole blood clots were used for targeting experiments. Human whole blood was drawn from healthy volunteers who signed the informed consent and did not take any medication at least two weeks before blood collection. The informed consent was approved by the ethical committee of the International Clinical Research Center St. Anne’s University Hospital Brno on 2017-03-07 (IIT/2016/30). Clots were prepared from 100 µL of whole blood without the addition of anticoagulants in glass tubes at room temperature for 4 h. The diameter of each glass tube for 100 µL of blood was 6 mm.

#### 2.3.2. Fibrin Clot

Fibrin clots were prepared from TISSEEL Kit Powders and Solvents for Sealant (Baxter, Deerfield, Illinois, USA) according to manufacturer’s instructions with slight modifications. Briefly, TISSEEL powder for sealant (containing fibrinogen, plasma fibronectin, factor XIII, and plasminogen) was reconstituted with 1 mL of distilled water. Aprotinin solution was replaced with 0.9% NaCl Solution. Then, 100 µL of resultant TISSEEL solution was mixed with 100 µL of bovine serum in 8 mm diameter glass tubes. After mixing, 100 µL of thrombin (500 I.U.) was added. The clot was removed from glass tubes after 1 h and was immediately used for experiments. For preclinical imaging, fibrin clots were prelabelled with corresponding contrast agents, as follows. For microCT, 30 µg of barium sulphate (Micropaque CT 50 mg/mL solution, Guerbet, France) was added to the TISSEEL solution prior to mixing with thrombin solution. Artificial fibrin clots for a rat middle cerebral artery occlusion (MCAO) model labelled by Micropaque were prepared as described above with a slight modification: 1.5 mL of saline solution and 0.5 mL of Micropaque were injected into TISSEEL original ampule. In the 500 I.U. thrombin original ampule, 1 mL of original CaCl_2_ solution and 5 mL of bovine serum were injected. Original Duploject system injected to PE60 cannula ana partes solution from TISSEEL and 500 I.U. thrombine was used.

For the purpose of FLI and MRI, 50 µL (1 mg/mL of total lipid) of the fibrin targeted double-labelled liposome solution was used for clot pre-labelling by adding liposomes directly to non-coagulated blood or fibrinogen solution (TISSEEL solution) before clotting or prior to mixing with thrombin solution, respectively.

#### 2.3.3. SEM of Fibrin Clot

The fibrin clot structure contrasted with barium sulphate was observed using a scanning electron microscope; Hitachi SU8010, Japan. Prior to observation, samples were fixed in Millonig phosphate buffered gluteraldehyde (3%), post-fixed in osmium Millonig buffered (OsO_4_ 2%) solution, dehydrated in 50, 70, 90, and 100% ethanol and dried in hexamethyldisilazane (HMDS, Sigma-Aldrich, Prague, Czech Republic). Samples of fibrin clots were put on the carbon tabs attached to the holder and platinum/palladium coated (Cressington Sputter Coater 208 HR, Watford, UK).

#### 2.3.4. Occlusion Formation in the 3D Printed MCA Model

The silicone MCA model was connected to a Lambda Multiflow pump (Lambda Laboratory Instruments, Baar, Switzerland) with 3.1 mm (inner diameter) tubes and filled with tris-buffered saline (TBS) buffer (pH 7.4). The prepared whole blood thrombus or fibrin thrombus was inserted into the tubing through a funnel to simulate the thromboembolic event in the MCA. Following the MCA occlusion formation, the flow rate through an arterial branch was 4.5 mL per minute. Fluorescein dye was added to observe the flow in the model and whether the occlusion caused by the clot was complete.

#### 2.3.5. Preparation of Gd- and Rhodamine-Containing Liposomes

Briefly, fibrin targeted metallochelation liposomes were prepared similarly to our previous work [19,20] and are described in detail in Supplementary Material S1.

### 2.4. In Vitro Clot Imaging Using Targeted Fibrin-Specific Liposomal Contrast Agent

Contrast-labelled clots were visualized using fluorescence/confocal microscopy, MRI and real-time FLI.

Microscopic techniques: the fluorescence microscope Nikon TE200 (Nikon, Tokyo, Japan) with Texas Red filter and a confocal microscope Leica TCS SP8 (Heidelberg, Germany) were used to detect D7H2 binder-functionalized rhodamine-labelled liposomes binding to fibrin filaments in the clot. Excitation and emission wavelengths were set to 561 nm and 575–650 nm, respectively.

Multispectral FLI: fluorescence imaging of whole blood clots labelled with D7H2 binders carrying rhodamine liposomes were performed using In-Vitro Xtreme 2 (Bruker Preclinical Imaging, Ettlingen, Germany). The silicone MCA model with a clot was placed into a tray. The MCA model was connected to the Lambda Multiflow pump (Lambda Laboratory Instruments) and placed inside the Xtreme 2. Fluorescence images were acquired using excitation and emission filters of 540 and 600 nm, respectively, with 8 s exposure and 2 × 2 binning. Reflectance images were acquired as background. The clots were imaged every 10 min with a 30 min time interval under a constant TBS buffer flow.

Magnetic resonance imaging (MRI): the gadolinium pre-labelled clots in the MCA model were imaged in a 9.4 T magnetic resonance system (BioSpin 94/30, Bruker BioSpin, Ettlingen, Germany) using a quadrature volume coil (inner diameter 86 mm) as follows: T1-weighted images were acquired using a standard spin–echo sequence with TR/TE = 700/11 ms ms (TR—repetition time, TE—echo time), averaging 3, matrix size = 256 × 256, field of view (FOV) = 55 × 50 mm and slice thickness 0.7 mm. Images used for quantification of T1 values were measured using a variable-TR rapid-imaging-with-refocused-echoes (VTR RARE) sequence with TR = 5500, 3000, 2000, 1200, 700 and 463 ms, TE = 7 ms, RARE factor 2, averaging 1, matrix size = 256 × 192 and the same geometry as for the T1-weighted images. T1 was quantified in the hand-drawn regions of interest using a curve fitting in the image sequence analysis (ISA) tool (ParaVision v.6.0.1, Bruker BioSpin, Ettlingen, Germany).

### 2.5. Determination of the Rate of Fibrinolysis Using MCA Model and MicroCT Imaging

The course of fibrinolysis of a fibrin clot occlusion in the MCA model was assessed by measuring the clot volume using microCT. After inserting the barium sulphate-labelled fibrin clot into the silicone MCA, the MCA was filled with TBS buffer containing 10% blood plasma and then connected to the pump. The silicone MCA was then placed into a large cylindrical scanning bed (diameter 7 cm) and scanned using microCT Skyscan 1276 for 160 min with a 20 min interval. The pump was placed on the heating plate (37 °C) outside the scanning machine and the flow was set to 4.5 mL/min. The scanning parameters were as follows: continuous scanning mode with 292 projections, binning 1 K, voltage 100 kV, current 20 µA, exposure time 147 ms, aluminum–copper filter, scan duration 46 s. The experiment started with rtPA injection. A concentration of rtPA relevant to clinical administration in patients with ischemic stroke was calculated according to manufacturer’s instructions (Actilyse; Boehringer-Ingelheim, Ingelheim am Rhein, Germany) and available pharmacokinetic data (Acheampong 2012). The final concentration of rtPA in circulating buffer was 1.3 mg/L. Occlusion site in the MCA model was monitored for 160 min after rtPA injection.

Back-projections of each time point interval were reconstructed using InstaRecon software (Bruker microCT, Kontich, Belgium) with ring artifact reduction 7.0 and no beam hardening correction, resulting in 551 slices with a final image pixel size of 852 × 348. Clot volume size in voxels was calculated using CTan software version 1.18.8.0 (Bruker microCT, Kontich, Belgium).

### 2.6. Middle Cerebral Artery Occlusion (MCAO) Rat Model

To verify the utilization of barium-labeled fibrin clots for microCT imaging in vivo, middle cerebral artery occlusion was induced in a single adult male Wistar rat according to [21] with slight modifications. Briefly, the left common carotid was gently isolated in its bifurcation to internal carotid (ICA) and external carotid (ECA). The thyroid and occipital artery were cauterized, the pterygopalatine artery was ligated. The ECA was ligated in the distal end, then cut and the PE50 catheter was inserted via ECA retrogradely into ICA. One piece of the artificial clot was injected into ICA. The experiment was approved by the national committee for animal protection at the Ministry of Agriculture of Czech Republic, No. MZe 2162.

### 2.7. Statistical Analyses

Statistical analyses of data from microCT and FLI were performed using GraphPad Prism version 8.3.0 (San Diego, USA). The rate of fibrinolysis was analyzed using two-way analysis of variance (ANOVA) where clot volumes in time were compared. One-way ANOVA was used to compare differences in fluorescent signals in FLI. Statistical tests were considered to be significant at *p* < 0.05.

## 3. Results

### 3.1. Quality Assessment of the Silicone Stenotic MCA Model

**Figure 2:**
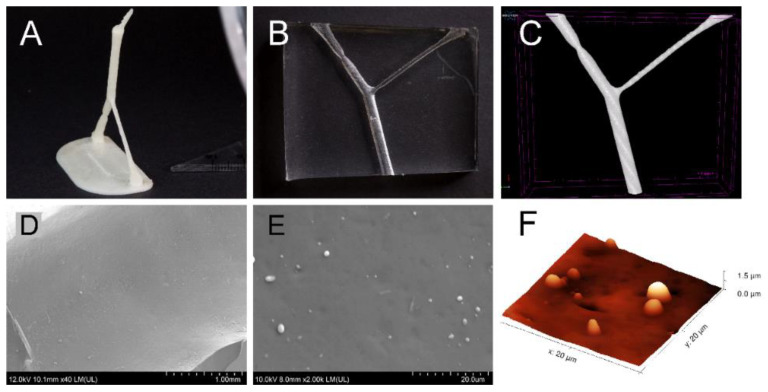
In vitro middle cerebral artery models prepared using 3D printing. A MCA model 3D printed using butenediol vinyl alcohol (BVOH) filament before casting into silicone (**A**), silicone MCA model prepared by dissolution of casted BVOH (**B**), 3D-rendered volume of the silicone MCA model obtained from microCT scanning showing the high quality of final MCA model without any imperfections (**C**), the surface of MCA lumen from silicone was observed using scanning electron microscopy. Only microscopic spherical-shaped imperfections of hundreds of nanometers were observed on the smooth surface of the silicone (**D**), detailed view of SEM image (**E**), AFM confirmed very smooth surface with hundreds of nanometer-sized blobs (**F**).

The quality of the MCA model (*n* = 10) was assessed using microCT, SEM and AFM (Figure 2) to verify the reproducibility of the model. No silicone deformation was observed after casting. After dissolving the MCA BVOH 3D print, the final silicone model was flexible and transparent. The shape of the final MCA silicone model was identical to the 3D printed BVOH MCA (Figure 2A–C). A 3D rendered volume of the silicone MCA model had also shown smooth lumen artery surface without visible artefacts (Figure 2C, video—Supplementary Material S2). SEM and AFM of the inner lumen surface showed a smooth surface (Figure 2). Only occasional blobs of sub-micron size at the artery lumen surface were observed (Figure 2D–F). This should provide a concise and precise description of the experimental results and their interpretation, as well as the experimental conclusions that can be drawn.

### 3.2. Simulation of Thromboembolic Stroke Using In Vitro Stenotic MCA Model

**Figure 3:**
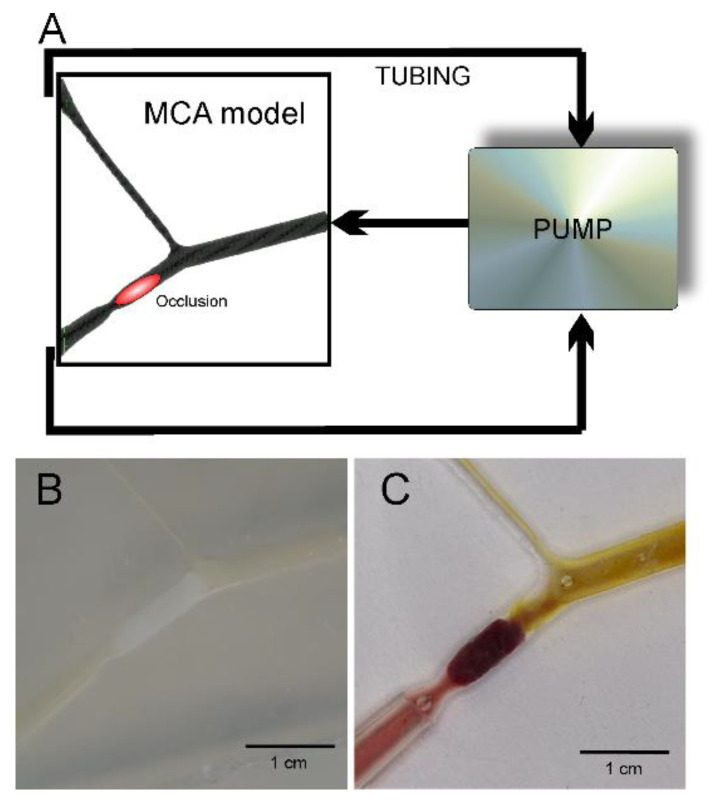
Complete occlusion of in vitro middle cerebral artery. (**A**) Schematic representation of the whole system composed of the pump, tubing, and the MCA model. Part of the MCA model with occlusion induced by fibrin clot prepared from the TISSEEL kit (**B**) and whole blood clot (**C**). Yellow fluorescein-colored plasma in incoming parts of the MCA and red blood cell-colored fluid behind the clot confirmed complete occlusion of the arteria lumen (**C**).

To document the ability of the model to simulate acute thromboembolic stroke, a clot was prepared externally and inserted into tubing in front of the silicone MCA model connected to a pump as shown in the schema (Figure 3A). Using both the fibrin clot (Figure 3B) or whole blood clot (Figure 3C), stable occlusion was observed in front of the stenosis in all experiments (*n* = 10) as shown in the demonstration video (Supplementary Material S3). Impermeability of the occlusion was observed and confirmed by the lack of fluorescein dye behind the occlusion as shown in Figure 2C. In addition, the lack of the flow through the clot site was observed during the experiment as documented by the presence of the backward swirling flow in front of the occlusion (Figure 3C). Occlusion was established at the same site of the silicone MCA model during all experiments performed.

### 3.3. In Vitro Clot Visualization in the Silicone Stenotic MCA Model Using Targeted D7H2 Liposomes

Previously developed D7H2 fibrin-specific protein binder was proven to bind to artificial fibrin clot prepared from the TISSEEL kit used in this study (Supplementary Material S4). Furthermore, D7H2 protein binder was used for construction of fibrin-targeted, dual-labelled liposomal contrast suitable for preclinical FLI and MRI of clots. Then, 245 nm-sized metallochelation liposomes with negative ζ-potential were prepared. D7H2 protein binder was bound onto the surface of liposomes, which was reflected by an increase in hydrodynamic radius of resultant D7H2 proteoliposomes from initial 245 nm (plain liposomes) to 255 nm (binder-modified liposomes) (see Supplementary Material, Figure S1).

Furthermore, liposomes modified by D7H2 were used for both targeting study (whole blood clot) and pre-labelling of thrombus for FLI and MRI imaging (fibrin clot).

**Figure 4:**
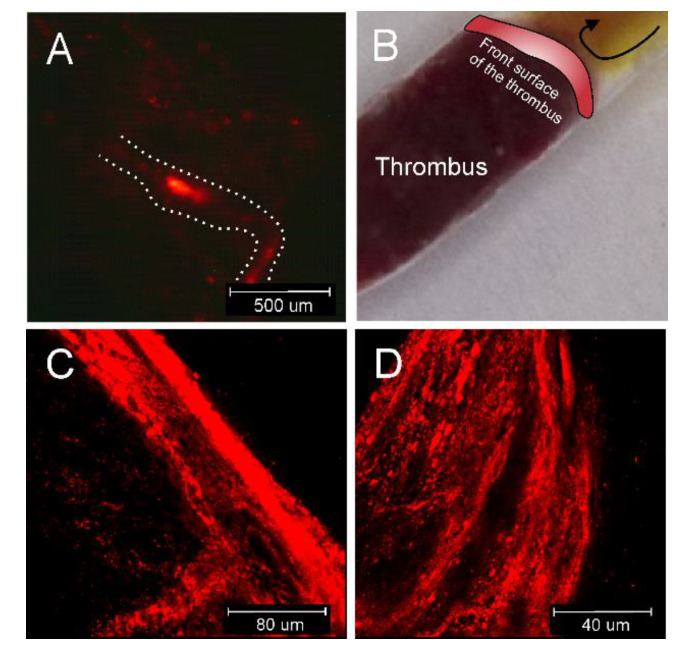
Targeting of fibrin filaments of the clot in the silicone MCA model under flow conditions visualized using fluorescent imaging. Fluorescent microscopic imaging of fluorescently-labelled liposomes (red) targeted to fibrin filaments of the clot in the MCA model. Focused plane of the front surface of thrombus is visualized inside MCA model (**A**). Schematic representation of the front area of the clot targeted by liposomes (red) under flow conditions (**B**). Confocal microscopy of the clot after extraction from the silicone model (cross-section). The front surface layer of the thrombus with highly targeted fibrin fibrils is clearly distinguishable from deeper layers with significantly lower number of liposomes using confocal microscopy. (**C**). Detail of cross-section (**D**).

To confirm the applicability of the 3D printed model for fibrin targeting studies, clots prepared from whole human blood were placed into the MCA. Fluorescent microscopy of the clot directly visualized inside the 3D printed MCAO model showed that only an adjacent relatively thin layer of the clot was targeted by circulating rhodamine-labelled D7H2 liposomes in the experiment (*n* = 3) (Figure 4A). Figure 4B represents the schema of the flow of liposome-containing buffer surrounding the front surface of the clot. Deeper layers of the clot were weakly targeted by rhodamine-labelled liposomes when compared to approximately 100 µm thin front layer as confirmed by confocal microscopy immediately after the removal of the clot from the MCA model (Figure 4C,D).

### 3.4. Thrombus Visualization in the Stenotic MCA Model Using Preclinical Imaging Techniques

**Figure 5:**
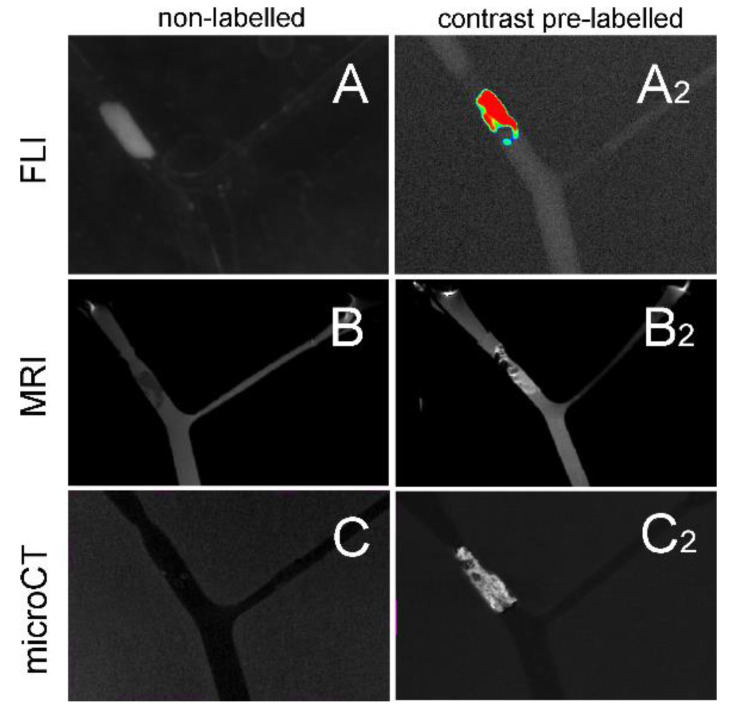
Imaging of pre-labelled clots in the MCA model using various preclinical imaging techniques. Multispectral fluorescent imaging (**A**), magnetic resonance imaging (MRI) (**B**), and microCT (**C**). Rhodamine-labelled, and gadolinium-labelled D7H2 liposomes targeted to fibrin were used for fluorescence imaging (FLI) and MRI, respectively. Non-targeted barium sulphate microparticles for microCT visualization were used as a contrast agent: negative controls (**A**–**C**), contrast pre-labelled clots (**A_2_**–**C_2_**). (**A**)—the signal of scattered light. Each experiment was repeated three times, the figure shows representative pictures.

Multispectral FLI, MRI and microCT techniques were explored for capability of visualization of contrast-prelabelled fibrin thrombi in our 3D printed MCA model. Both targeted and non-targeted contrast agents were explored for clot imaging in this study. We confirmed intensive contrast signal of the clot by multispectral FLI and MRI imaging using D7H2-targeted liposomal contrast (Figure 5A,B). Real-time FLI of rhodamine liposome-labelled clots in the 3D printed MCA model revealed the presence of fluorescent signals in the site of the clot (Figure 5A). During the constant flow, intensities recorded in 10 min intervals for 30 min did not differ significantly (*p* > 0.05).

Similarly, fibrin clots labelled with targeted gadolinium liposomes placed in the 3D printed MCA model and imaged by magnetic resonance were clearly visible and resulted in a brighter signal in T1-weighted images compared to the control clot (Figure 5B). Quantitatively, T1 measured within the labelled versus control clots was 1251 ± 26 versus 2452 ± 60 ms, corresponding to the expected T1 shortening due to Gd-liposomes. The non-targeted contrast agent, barium sulphate, was employed for microCT imaging (Figure 5C). Observed average clot radiodensities were 130 ± 34 and −18 ± 41 Hounsfield units in barium sulphate-pre-labelled and non-labelled clots, respectively.

### 3.5. Monitoring of Thrombolysis under Flow Conditions in the Stenotic MCA Model Using MicroCT

In this study, microCT was selected from the group of preclinical imaging techniques (FLI, MRI, CT) to prove the concept of precise monitoring of fibrinolysis in vitro.

**Figure 6:**
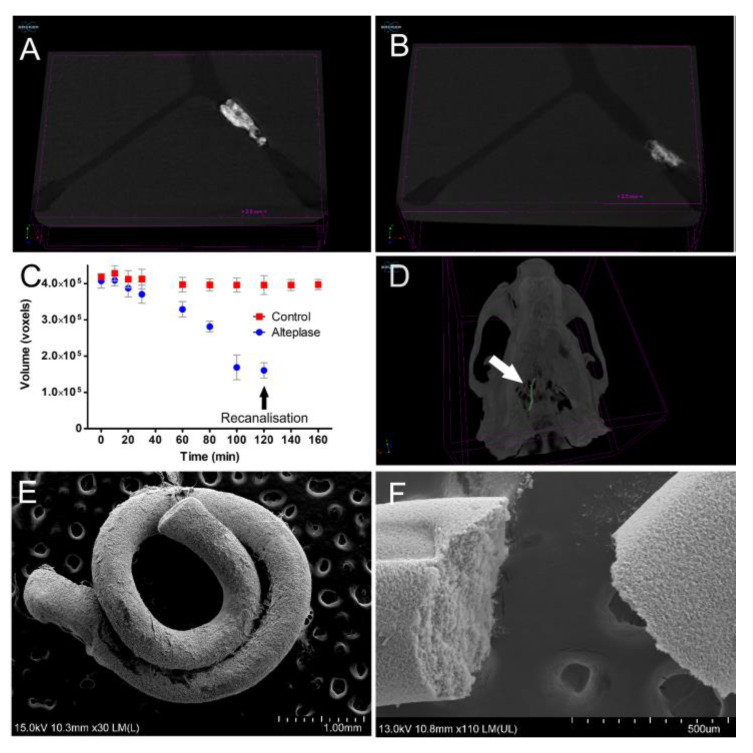
Re-canalization of the MCA model observed using microCT. Barium sulphate was used as a contrast agent for visualization of the thrombus in the MCA model. Visualization of the thrombus at time zero (**A**), and after recombinant tissue plasminogen activator (rtPA) treatment at 120 min (**B**). The volume of the thrombus as a function of time during 160 min time interval after rtPA treatment analyzed by GraphPad Prism from microCT pictures: a negative control (red square), rtPA treated (blue circle) (**C**). Proof-of-concept of barium sulphate thrombus labelling under in vitro conditions. The experiment demonstrated the application of microCT for both in vitro and in vivo barium sulphate-labelled thrombus imaging; thus, serving for precise in vitro and in vivo thrombolysis monitoring. The rat’s brain artery at time zero was visualized using pre-labelled thrombus (**D**). SEM image of pre-labelled thrombus prepared using the TISSEEL kit applied to rat brain (**E**). Detail of the structure of fibrin thrombus (**F**).

The 3D printed MCA model enabled similar flow conditions to that occurring during stroke in vitro. Therefore, studying the thrombolytic effects induced using various thrombolytics on standardized models is an advantageous approach. In our experiment, fibrin clots labelled with barium sulphate were exposed to rtPA in the silicone MCA model (Figure 6A,B). Three-dimensional rendering volume models of rtPA exposed clots in time showed an obvious decreasing trend in a clot’s volume in time. Recanalization time for fibrin clots using microCT evaluation was 120 min (Figure 6C). The volume of the thrombus at time 0 significantly differs from those recorded at time 120 min, where 42% of the monitored clot volume remained after 120 min (Figure 6C, blue circles). The volume of control fibrin clot did not change in time (160 min) (Figure 6C, red squares). We observed no effect of incorporated barium sulphate in the clot on the rate of clot dissolution (Supplementary Material S5).

To confirm the applicability of barium sulphate-labelled clots for imaging in vivo, a standard MCAO rat model was employed. MicroCT scan of clot injected into the rat circle of Willis revealed the presence of highly contrasting clots (radiodensity higher than bone) in rat brain (Figure 6D). The porous structure of sulphate barium contrasted fibrin clots with the clear structure of fibrin fibrils used for in vitro experiments was observed by SEM (Figure 6E,F).

## 4. Discussion

In the present study, we showed that a 3D printed silicone MCA flow model in combination with advanced preclinical imaging techniques is a useful tool for assessment of the effectivity of direct thrombus targeting as well as a valuable tool for evaluation of thrombolytic activity in vitro. To validate this model, we used advanced preclinical imaging techniques with CT being used for stroke patients’ imaging and thus making our model relevant for translational research. Our model, due to its high throughput, can be used as the first line of screening and can speed up the development and avoid excessive animal testing in preclinical research. Due to presence of the flow, the silicone MCA model possesses realistic conditions during the experiment. Furthermore, a novel method for in vitro evaluation of thrombolytic activity using microCT and barium sulphate-labelled fibrin clots was shown in this study.

**Scheme 1:**
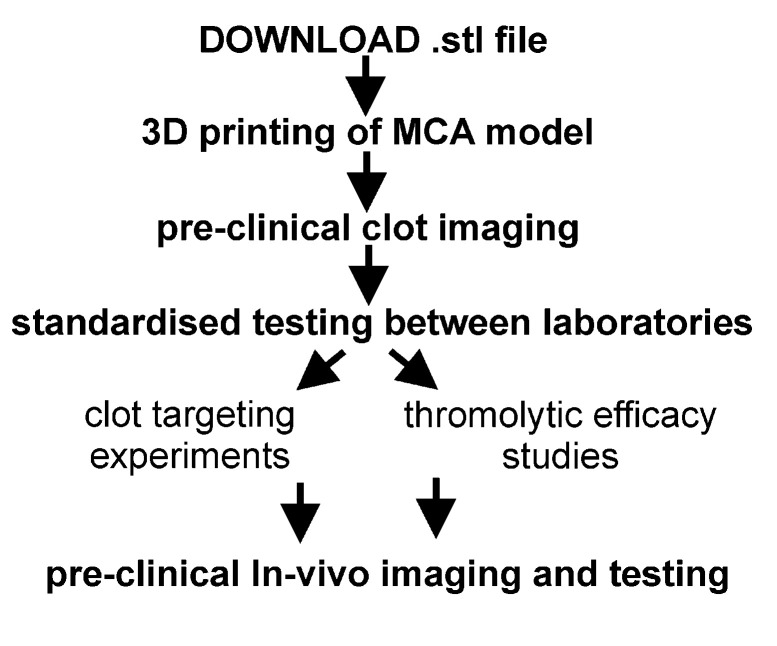
An approach to standardize the in vitro testing between laboratories in the area of stroke research, employing 3D printing and clot imaging.

Furthermore, 3D printing has become broadly available and can be utilized in various in vitro models [22]. Here, we show the application of 3D printing for the preparation of a highly standardized silicone MCA model. The presented model can be easily reproduced in other laboratories worldwide with a high level of precision. Therefore, printable files (.stl format) of the in vitro model are provided alongside the publication as part of the Supplementary Materials (S6) to enable cheap and fast reproduction of the MCA model. Such a strategy has a great potential to contribute to the standardization of experiments across laboratories and contributes to using this simplified model as well as more sophisticated models including replicas of patient blood vessels (Scheme 1).

A similar approach of model fabrication of blood vessels was reported by N. Kaneko, where they reported fabrication of patient-specific silicone vessels employing acrylonitrile butadiene styrene [23]. Patient-specific vessels were intended for the training of physicians for minimally invasive endovascular procedures in this study [23]. Similarly, Ruth et al. used a 3D printed model of middle cerebral artery aneurysm for neurosurgery simulations [24].

The in vitro 3D printed silicone model was designed to anatomically correspond to the diameter of arteries, bifurcation, and to enable permanent flow such as most cases during ischemic stroke [25]. The design of the model thus enables the simulation of thromboembolic stroke in stenotic MCA M1 segment but also, due to its simplicity, enables the extraction of the clot from the model without damage to enable further clot study testing and characterization in various time points of experiments performed (e.g., morphological studies of the clot using SEM/cryo-SEM, confocal microscopy studies (Figure 4), etc.). Analysis performed using preclinical imaging techniques (Figure 5) can be thus combined with exact micro-to-nanoscale analysis of clot structure (Figure 6E), various functional analyses using antibody staining (e.g., Figure S3), AFM imaging and other advanced techniques. Despite several advantages of the 3D printed model present in this study, the main limitations include lacking the rest of the circle of Willis to perfectly mimic blood vessels in the brain, and also the flow rate and pressure do not exactly correspond to the physiological conditions.

In order to provide full characterization of the model, the resultant MCA model was visualized using microCT (Figure 2C), showing a smooth surface. To observe the internal surface in high resolution, SEM and AFM techniques were applied. The surface of the silicone was observed to be smooth with only micro-meter-sized imperfections (Figure 2E,F). Therefore, we suppose no significant effect on the reproducibility of the fluid flow during the experiments.

In our experiments, externally prepared clots were inserted into the MCA model. Using the mild flow, clots were anchored in the stenosis of the tubing before each experiment. Various types of clots have been used for clot targeting and thrombolytic studies in vitro, each having different mechanical properties, and different fibrin and blood cell content [16,26,27,28].

In our study, we proved the feasibility of experiments using both whole-blood clots and fibrin clots (TISSEEL kit) inserted in the MCA model. Whereas whole-blood clots were used for fibrin targeting experiments, fibrin clots (TISSEEL kit) were employed for imaging and fibrinolytic studies. The reason for using fibrin clots (TISSEEL kit) for fibrinolytic studies is that fibrin clots were selected for further in vivo testing due to its mechanical properties, which are crucial for stable MCA occlusion formation in rat models (Figure 6D), and thus transferability and comparability of in vitro and in vivo data in follow-up experiments. On the other hand, whole-blood clots have more similar properties to the real clots occurring inside the blood vessels.

Our study holds promise for advancing clinical research due to several reasons. Firstly, clot imaging is powerful tool to directly measure thrombolytic activity in in vitro and in vivo experiments. Such a tool can accelerate research of novel thrombolytics and recanalization strategies in general, which are highly needed in clinical practice due to limited recanalization properties of currently used thrombolytics. Additionally, our models can allow better understanding of how different qualities of the clot and/or hemodynamic parameters influence efficacy of current thrombolytic treatments. Better knowledge is needed, especially because several clinical studies have suggested that clot length, clot permeability, and clot histopathology interact with each other and could explain different levels of susceptibility of clots to lysis. Finally, clinicians may benefit from direct thrombus imaging due to easy and precise clot localization in patients in contrast to standard indirect thrombus imaging using angiographic techniques or ultrasound [9,29,30].

We show various possibilities of clot imaging in a 3D printed MCA model using both targeted and non-targeted contrast agents for FLI, MRI, and CT (Figure 5). All imaging techniques are applicable to preclinical animal models, and thus the information gained from the experiments using standardized in vitro models could be extremely important, and finally lead to reduction in the number of animals needed.

The applicability of the 3D printed MCA model for direct thrombus imaging studies was performed using D7H2-modified fibrin-targeted liposomes. In this study, we formulated D7H2-proteoliposomes in the Z-average size of 250 nm, dual-labelled using lyssamine-rhodamine, and Gd-PE for FLI and MRI, respectively. D7H2 protein was previously reported to bind to clots prepared from human whole blood [31]. Here, we also proved the specific binding to artificial clots prepared from the TISSEEL kit (Figure S3). Therefore, dual-labelled D7H2-modified liposomes were employed for both clot targeting (whole blood clots), and also clot pre-labelling and consequent imaging using FLI and MRI (artificial fibrin clots). Modification of liposomes using D7H2 and its physical chemical characterization was described in detail previously [31].

The clot targeting study in vitro was performed using whole-blood clots under flow conditions, with total occlusion of the stenosis. As a result, massive targeting of the front surface layer of the clot of a thickness of about 100 um was observed when compared to deeper layers of the clot (Figure 4), indicating that both factors probably manifest (i) the affinity of D7H2-modified liposomes to fibrin fibrils, and (ii) the relatively slow diffusion of nanoparticles into the structure of the fibrin mesh, influenced by phenomena of hydrodynamic diffusion suppression, and steric diffusion suppression [32], both leading to observed effect in this study. However, the effect of various flow rates on the depth of liposome penetration into the clot needs to be further studied as higher flow rate and pressure naturally occurring in the MCA could have a positive effect on the depth of the liposome penetration. Clot perviousness, associated with densities of major components including red blood cells, white blood cells, fibrin, and platelet conglomerates, plays an important role in responsiveness to therapy. Higher red blood cells content and lower fibrin density are associated with higher clot perviousness [33]. Similarly, permeability of clots would probably have an impact on the efficacy of clot imaging using nanoparticle-based targeted contrast agents.

In contrast to clot targeting under flow conditions, pre-labelling of fibrin clots using D7H2 liposomes produced homogenous distribution of signals when imaged using FLI, and MRI (Figure 5A_2_,B_2_). Therefore, the experimental setup using contrast pre-labelled clots and consequent quantification of the clot volume/intensity of the signal from a contrast agent has a great potential for advanced in vitro thrombolytic studies. The homogeneity of the signal is given due to the fact that clots were formed from a homogenized solution of fibrinogen and dual-labelled fibrin-targeted liposomes. Within all clot imaging studies, as well as fibrinolytic studies, pre-labelled fibrin clots were employed to get optimal signals from clots, enabling precise quantification of the signal obtained, leading to real-time information on the clot volume. Fibrin targeted contrasting MRI imaging has already been used in various models [8], including a rabbit model [34,35]. In the present study, however, a novel fibrin binding system using protein binders and liposomal carriers [31] with gadolinium-labelled contrast agent was applied [36].

This contrast agent for direct thrombus MRI imaging looks promising in vitro; however, sensitivity and specificity need to be further verified in animal models, and further optimization for animal models are needed.

In this series of experiments, we found that all tested approaches of clot pre-labelling and preclinical imaging techniques using a 3D printed in vitro flow model give sufficient contrast intensity, and thus are feasible to perform. It is worth noting here that all mentioned imaging techniques are applicable for in vivo experiments, and thus are easily transferable from in vitro flow models to animal experiments, and finally leading to decreased number of experimental animals needed.

In animal studies, recanalization after rt-PA therapy is affected not only by animal species but also by intraspecific variability [37,38]. In comparison to in vivo models, the absence of metabolism and inter- and intra-specific animal variability make the in vitro flow model an efficient tool for novel thrombolytic drug pre-screening.

Here, we have proven that most accessible animal preclinical imaging techniques including FLI, MRI, and CT are also potentially applicable for in vivo assessment of (i) clot targeting efficacy, and (ii) thrombolytic/fibrinolytic effects of potential drugs or therapeutic approaches (Figure 5). Moreover, we have shown that barium-labelled clots can be imaged with sufficient resolution both in vitro and in vivo making microCT extremely precise and an efficient tool for evaluation of thrombolytic activity (Figure 6).

Quick microCT scans of the clot offer clot volume determination in time with a precise resolution of around 40 µm. This clot volume reduction in time allows dynamic evaluation of clot lysis. Importantly, barium sulphate particles did not affect the thrombolytic process, as was documented in our in-tube clot lysis trials with and without barium sulphate. In addition, barium provides an excellent contrast both in vitro and in vivo (Figure 6). There is, thus, a strong potential for utilization of barium-labelled fibrin clots for in vivo preclinical models of novel thrombolytic therapies.

Time needed to remove the fibrin clot from the stenosis site (recanalization time) lasted approximately 120 min in our experiments using a 3D printed flow model in vitro. It is almost impossible to compare recanalization time between various in vitro or in vivo models due to the many factors involved. In principle, an advantage of the 3D printing method for production of the MCA model presented in this study is a high level of standardization across laboratories, due to the ease of sharing printable files and low-cost of production. Thrombolysis assessment in our in vitro flow MCA model may enable testing of novel thrombolytic agents under well-defined conditions, including standardized fibrin clot (the same source of fibrinogen, the same size), standardized plasma dilution, flow, and temperature etc. In addition, thromboembolic occlusion simulated in artificial stenosis in the silicone MCA better reflects the situation where the clot is lysed only from an afferent area of the vessel.

## 5. Conclusions

In conclusion, the 3D printed transparent flow MCA model is applicable for the prediction of thrombolytic effects as well as contrast density of new thrombolytics and theranostics before testing is performed in preclinical animal models. It involves various studies performing clot imaging, including the determination of the efficiency of clot targeting and the efficiency of various thrombolytic approaches including novel fibrinolytic drugs.

The 3D printing technology used represents a great potential to standardize experiments across laboratories as 3D printing is a broadly accessible, easy, and cheap method for in vitro model production. FLI, MRI and microCT were proven to be effective preclinical imaging techniques applicable for clot imaging introduced into a 3D printed transparent flow MCA model. The simplicity of the model enables the clot extraction from the MCA model at various stages of the experiment, and thus enabling a combined use of preclinical imaging techniques with consequent clot analysis using advanced techniques including SEM, confocal microscopy, AFM, and others.

Moreover, in this study, we have proven for the first time that the non-targeted contrast agent barium sulphate is a suitable contrast for fibrinolytic studies in vitro using a 3D printed MCA model. Moreover, barium sulphate also has great potential for animal preclinical testing, as shown on a rat model. Finally, utilizing preclinical imaging techniques combined with a 3D printed MCA model can contribute to reduced animal experiments and experiment standardization.

## Supplementary Materials

The following are available online at https://www.mdpi.com/1999-4923/12/12/1207/s1, Supplementary Material S1: Preparation and characterization of Gd- and Rhodamine-containing liposomes, Video S2: Quality assessment of the silicone MCA model using microCT, Video S3: Simulation of thromboembolic stroke using in vitro MCA model, Supplementary Material S4: Targeting of fibrin fibrils of fibrin clot prepared from TISSEEL Kit using D7H2 protein binder, Supplementary Material S5: The effect of barium sulphate addition in the clot on clot lysis rate.
