# Supplementary material for: Thrombus Imaging Using 3D Printed Middle Cerebral Artery Model and Preclinical Imaging Techniques: Application to Thrombus Targeting and Thrombolytic Studies
Source: Pharmaceutics. 2020 Dec 12;12(12):1207. doi: 10.3390/pharmaceutics12121207 (PMC7763938; doi:10.3390/pharmaceutics12121207)
Supplement: Supplementary file 1 [file pharmaceutics-12-01207-s001.zip › S1.docx]

Supplementary Material S1: Preparation and characterization of Gd- and Rhodamine-containing liposomes

Thrombus Imaging Using 3D Printed Middle Cerebral Artery Model and Preclinical Imaging Techniques: Application to Thrombus Targeting and Thrombolytic Studies

Andrea Vítečková Wünschová, Adam Novobilský, Jana Hložková, Peter Scheer, Hana Petroková, Radovan Jiřík, Pavel Kulich, Eliška Bartheldyová, František Hubatka, Vladimír Jonas, Robert Mikulík, Petr Malý, Jaroslav Turánek and Josef Mašek

Materials and methods

All phospholipids were purchased from Avanti Polar Lipids: EPC (egg phosphatidyl choline), LissRhod-PE (1,2-dioleoyl-sn-glycero-3-phosphoethanolamine-N-(lissamine rhodamine B sulfonyl), PE-DTPA(Gd) (1,2-distearoyl-sn-glycero-3-phosphoethanolamine-N-diethylenetriaminepentaacetic acid (gadolinium salt)), Cholesterol, DGS-NTA(Ni) (1,2-dioleoyl-sn-glycero-3-[(N-(5-amino-1-carboxypentyl)iminodiacetic acid)succinyl] (nickel salt)), DSPC (1,2-distearoyl-sn-glycero-3-phosphocholine). The liposomes were prepared using phospholipid film hydration method and were extruded through the 200 nm Milipore filter using a hand extruder at 60 °C.

The composition of the dual-labelled liposomes was as follows: DSPC/PE-DTPA(Gd)/cholesterol/ LissRhod-PE /DGS-NTA(Ni) (48/20/20/5/7 *w/w/w/w/w*). Lipid film was hydrated with TBS to a final concentration of 20 mg/ml.

Surface modification of liposomes by fibrin-targeting protein binders

Surface modified metallochelatation liposomes were prepared as described previously (Mašek et al., 2011b, 2011a). The liposomes were mixed at room temperature in weight ratio anti-fibrin protein binder/total lipid 1/20 in TBS buffer. The mixture was stirred for 60 minutes.

Liposomes characterization

Measuring of size and ζ-potential of Gd and Rh liposomes

The size and ζ-potential were measured by dynamic light scattering on a Zetasizer ZSP (Malvern, UK). The measurements on the Zetasizer Nano ZSP were conducted in a quartz cuvette ZEN 2112. Cuvette DTS1060 was used for ζ-potential measurement. Both size and ζ-potential were measured at a temperature of 25°C and attenuator 7, at an angle of 175° (back scatter). The size and ζ-potential were measured at a total lipid concentration of 1 mg/ml in TBS, 10 mM Na-phosphate, pH 7.2.

Results


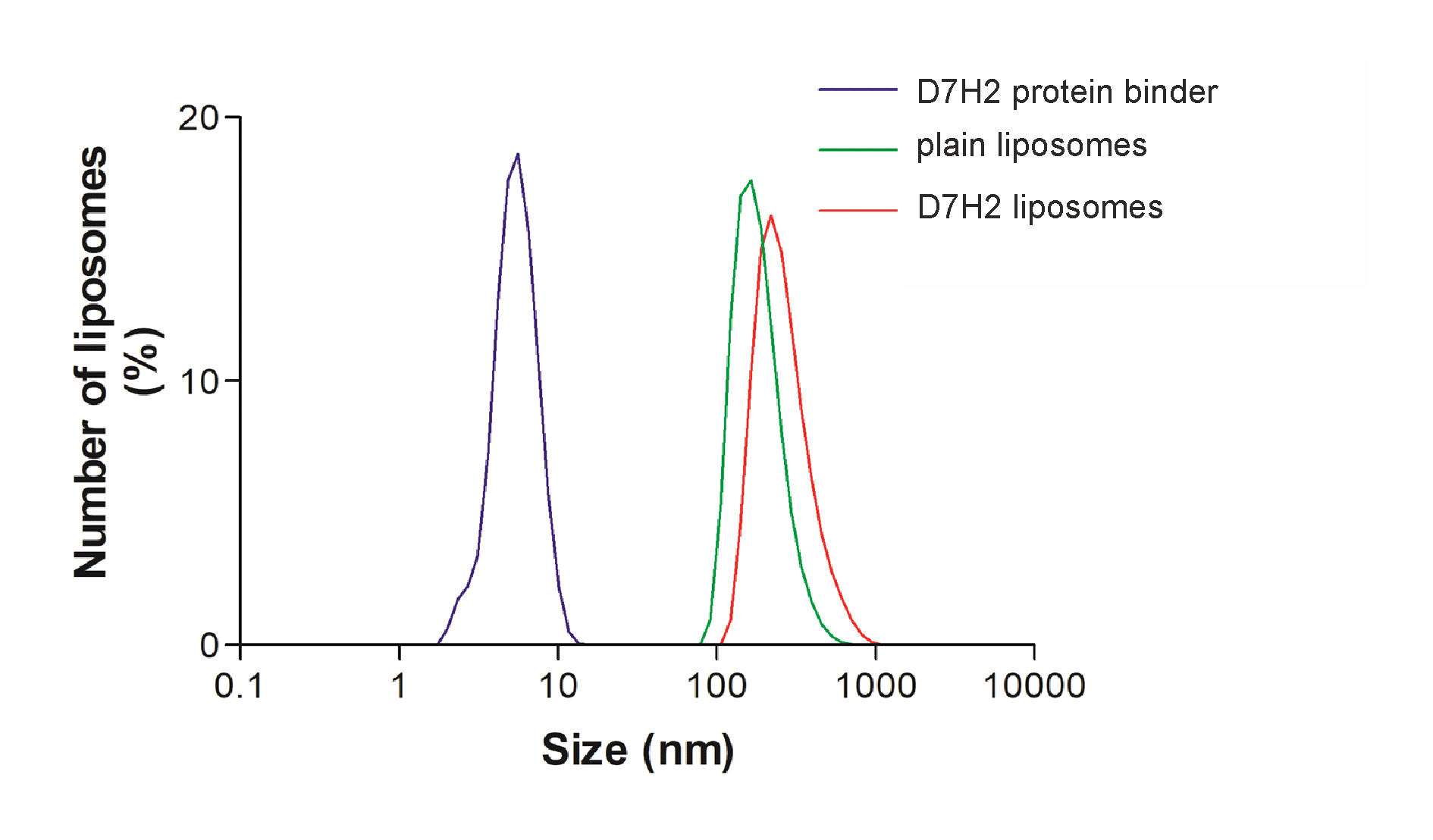


**Figure S4.** Hydrodynamic radius of D7H2 fibrin-specific protein binder, plain liposomes and D7H2 liposomes.

**Table 4.** Z-average (Z-ave) and polydispersity index (PDI) of D7H2 fibrin-specific protein binder, plain liposomes and D7H2 liposomes.

| **Sample** | **Z-Ave (nm)** | **PDI** |
| --- | --- | --- |
| D7 H2 | 4.5 ± 0.2 | - |
| plain liposomes | 245 ± 2.4 | 0.121 |
| D7H2 liposomes | 255 ± 3.5 | 0.132 |
