# Supplementary material for: Thrombus Imaging Using 3D Printed Middle Cerebral Artery Model and Preclinical Imaging Techniques: Application to Thrombus Targeting and Thrombolytic Studies
Source: Pharmaceutics. 2020 Dec 12;12(12):1207. doi: 10.3390/pharmaceutics12121207 (PMC7763938; doi:10.3390/pharmaceutics12121207)
Supplement: Supplementary file 1 [file pharmaceutics-12-01207-s001.zip › S4.docx]

Supplementary material S4. Targeting of fibrin fibrils of fibrin clot prepared from TISSEEL Kit using D7H2 protein binder

Thrombus Imaging Using 3D Printed Middle Cerebral Artery Model and Preclinical Imaging Techniques: Application to Thrombus Targeting and Thrombolytic Studies

Andrea Vítečková Wünschová, Adam Novobilský, Jana Hložková, Peter Scheer, Hana Petroková, Radovan Jiřík, Pavel Kulich, Eliška Bartheldyová, František Hubatka, Vladimír Jonas, Robert Mikulík, Petr Malý, Jaroslav Turánek and Josef Mašek

The capability of previously developed D7H2 fibrin-specific protein binder to target the artificial fibrin clot prepared from Tisseel Kit was tested. Followed fluorescent anti-HisTag staining, the intensive fluorescence signal was clearly visible on fibrin fibrils prepared from Tisseel Kit incubated with D7H2 protein as compared to the negative control. Brightfield pictures confirmed that the focus was in the desired plain (Figure S4).


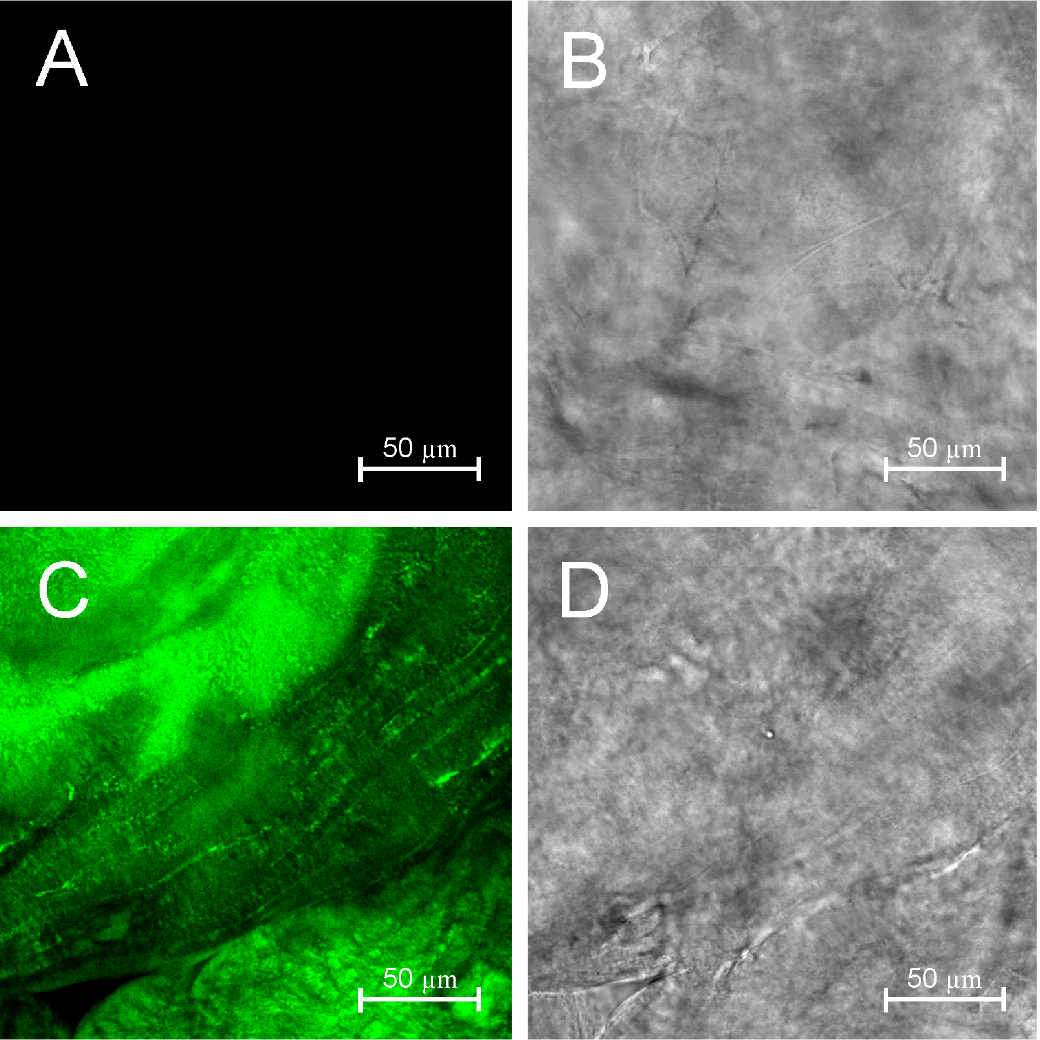


**Figure S4.** Targeting of fibrin fibrils of experimental fibrin-clot prepared using TISSEEL Kit. (**A**) negative control, AlexaFluor 488-labelled anti-HisTag antibody, (**B**) fibrin fibrils visualised in bright field of picture (**A, C**) visualisation of fibrin fibrils of clot using protein binder and AlexaFluor 488-labelled anti-HisTag antibody, (**D**) fibrin fibrils visualised in bright field of picture C.
