# Supplementary material for: Thrombus Imaging Using 3D Printed Middle Cerebral Artery Model and Preclinical Imaging Techniques: Application to Thrombus Targeting and Thrombolytic Studies
Source: Pharmaceutics. 2020 Dec 12;12(12):1207. doi: 10.3390/pharmaceutics12121207 (PMC7763938; doi:10.3390/pharmaceutics12121207)
Supplement: Supplementary file 1 [file pharmaceutics-12-01207-s001.zip › S5.docx]

Supplementary Material S5: The effect of barium sulphate addition in the clot on clot lysis rate

Thrombus Imaging Using 3D Printed Middle Cerebral Artery Model and Preclinical Imaging Techniques: Application to Thrombus Targeting and Thrombolytic Studies

Andrea Vítečková Wünschová, Adam Novobilský, Jana Hložková, Peter Scheer, Hana Petroková, Radovan Jiřík, Pavel Kulich, Eliška Bartheldyová, František Hubatka, Vladimír Jonas, Robert Mikulík, Petr Malý, Jaroslav Turánek and Josef Mašek

To determine the effect of barium sulphate addition into clots on clot lysis rate, in-tube lysis test using recombinant tissue plasminogen activator (rtPA) (Actilyse, Boehringer Ingelheim, Germany) were applied. Barium sulphate-labelled and non-labelled artificial fibrin clots were exposed to rtPA and clot lysis rate were evaluated using clot weight analysis.

Clot incubation and weight loss determination

In total of 18 artificial fibrin clots (9 barium sulphate labelled and 9 non-labelled colts) were used in the experiment. Each clot was gently washed with 2 ml of PBS buffer to remove serum residua. After removal of excess liquid using cotton wool pad for 10 s, individual clot weight were determined. Clots were then individually incubated in 500 µl of PBS containing 1.3 mg/l rtPA (Acheampong and Ford 2012) in a 1.5 ml plastic tube (Eppendorf, Germany) at 37°C for 60 min. Immediately after incubation, clots were blotted dry and weight was determined as described above. The weight loss (mean mass decrease) was as relative value of the difference between these two measurements to the initial mass.

**Results**


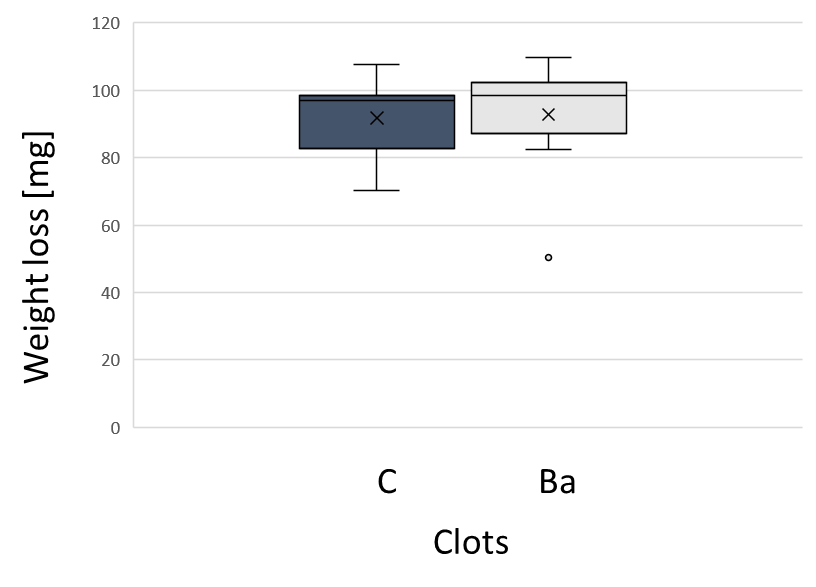
.

**Figure S5.** The effect of barium sulphate addition into clot on clot lysis rate. Control clots - without barium sulphate (**C**), Clots containing barium sulphate (**Ba**). Box plot: box is drawn between the first and third quartiles, with an additional line drawn along the second quartile to mark the median, × is mean and whiskers indicate variability outside the upper and lower quartiles, and any point outside those lines or whiskers is an outlier.

Conclusion

Control clots without barium sulphate addition (C) and clots with addition of barium sulphate showed no difference in weight loss during thrombolysis using rtPA.

Reference

Acheampong P.,Ford A.G. Pharmacokinetics of alteplase in the treatment of ischaemic stroke, Expert Opin Drug Metab Toxicol. 2012 Feb;8(2):271–281 doi:10.1517/17425255.2012.652615.
